# Human‐Aided Movement of Viral Disease and the Archaeology of Avian Osteopetrosis

**DOI:** 10.1002/oa.2599

**Published:** 2017-06-30

**Authors:** B. Tyr Fothergill

**Affiliations:** ^1^ School of Archaeology and Ancient History University of Leicester Leicester UK

**Keywords:** animal husbandry, avian leucosis virus, avian osteopetrosis, palaeopathology, Western Europe

## Abstract

The term avian osteopetrosis is used to describe alterations to the skeletal elements of several species of domestic bird, most typically the chicken, Gallus gallus *domesticus* (L. 1758). Such lesions are routinely identified in animal bones from archaeological sites due to their distinctive appearance, which is characterised by proliferative diaphyseal thickening. These lesions are relatively uncomplicated for specialists to differentially diagnose and are caused by a range of avian leucosis viruses in a series of subgroups. Only some avian leucosis viruses cause the development of such characteristic lesions in osteological tissue. Viraemia is necessary for the formation of skeletal pathology, and avian osteopetrosis lesions affect skeletal elements at different rates. Lesion expression differs by the age and sex of the infected individual, and environmental conditions have an impact on the prevalence of avian leucosis viruses in poultry flocks. These factors have implications for the ways in which diagnosed instances of avian osteopetrosis in archaeological assemblages are interpreted. By integrating veterinary research with archaeological evidence for the presence of avian leucosis viruses across Western Europe, this paper discusses the nature of these pathogens, outlines criteria for differential diagnosis, and offers a fresh perspective on the human‐aided movement of animal disease in the past through investigation of the incidence and geographic distribution of avian osteopetrosis lesions from the first century BC to the post‐medieval period. © 2017 The Authors International Journal of Osteoarchaeology Published by John Wiley & Sons Ltd.

## Dedication

This work owes an intellectual debt to the pioneering research of Don Brothwell, whose seminal paper on avian osteopetrosis in archaeological assemblages from England highlighted the potential interpretive value of these lesions (Brothwell, 2002). Professor Brothwell passed away during the preparation of this paper, which is dedicated to his memory.

## Introduction

Viral diseases of domestic animals impacted past human communities in often severe ways. Rinderpest (cattle plague, RPV) and swine fevers (CSFV and ASFV) have devastated herds in the last centuries. Beyond the threat to livelihoods, the former is likely the evolutionary source of the measles virus in humans (MeV) (Furuse *et al.,* 2010), and zoonotic viruses are a serious human health threat (Murray *et al.,* 2016). Archaeological evidence for viral infections in livestock is rare, and neither rinderpest nor swine fevers can be detected in skeletal remains. Although poultry are ‘household livestock’ and therefore not ideal models for viral transmission between cattle or caprines and humans, avian leucosis viruses (ALVs) (archaeologically visible as avian osteopetrosis) present opportunities to differentially diagnose and interpret macroscopic lesions resulting from infection by a known group of viruses, and contextualise viral disease in domestic animals in the past.

**Figure 1:**
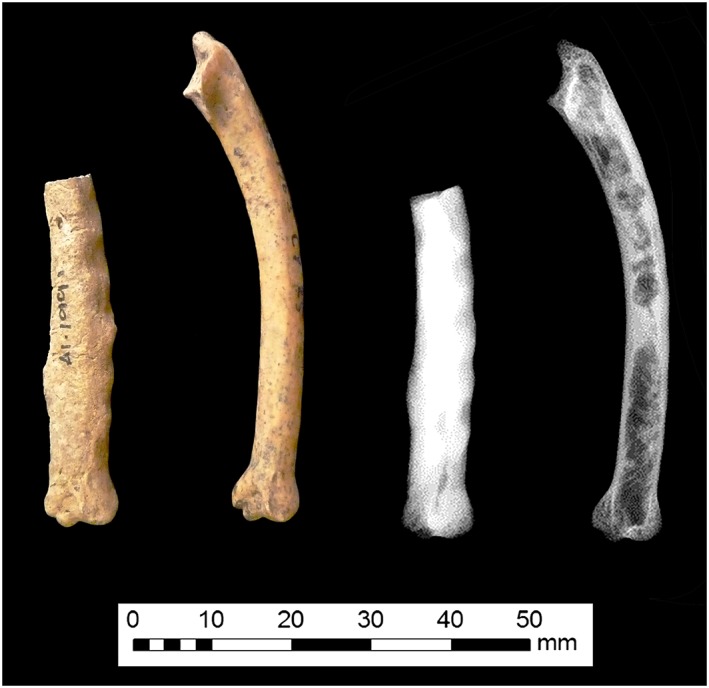
Osteopetrotic chicken ulna excavated from Causeway Lane, Leicester, shown with a comparative element. [Colour figure can be viewed at wileyonlinelibrary.com]

Non‐human palaeopathology specialists routinely analyse disarticulated, incomplete, and fragmentary skeletal remains, and the nature of these assemblages can prevent the advancement of interpretations beyond simple description and broad nosological classification. Pathologies which can be differentially diagnosed from a single element or partial specimen are of particular utility in interpreting relationships between human and non‐human animals in the past. Avian osteopetrosis, caused by ALV infection, is characterised by a distinctive lesion affecting osseous tissue (Figure 1) and has been included in animal palaeopathology manuals since the first major volume on the subject was published (Baker & Brothwell, 1980, 61–62). These lesions and their aetiology continue to be discussed in major works on palaeopathology (Bartosiewicz & Gál, 2013; Thomas, 2012). In addition to Brothwell's article (Brothwell, 2002), avian osteopetrosis lesions have been identified in a wide range of European archaeozoological investigations over the last few decades (Allison, 2010; Baker, 1998; Fabiš, 1997; Fothergill & Best, In press; Gál, 2008; Gál & Kunst, 2014; Gordon, 2016; Lentacker *et al.,* 2004; Luff & Brothwell, 1993; Morel, 1991; Peters, 1998; Prummel, 1987; van Wijngaarden‐Bakker & Krauwer, 1979; von den Driesch & Pöllath, 2000).

In noting the presence of avian osteopetrosis in an assemblage, the appearance of affected elements is customarily described and occasionally photographed, but discussion is rare and either limited to concepts such as stocking density or the spread of disease (Gál, 2008; Thomas, 2012) or supplied as an explanation for the disposal of an individual (Groot, 2008: 108). Furthermore, statements regarding the species vulnerable to infection by ALVs, the impact of the infection on soft tissue, which age groups and sexes of birds are susceptible, and the order in which elements across the skeleton are affected are presented with few references to the veterinary research (e.g. Albarella *et al.,* 2009; Fabiš, 1997).

This paper sheds new light on avian osteopetrosis and discusses the viruses which cause these characteristic lesions. It will examine: issues of problematic nomenclature and terminology; differential diagnosis of archaeological material; variation in lesion progression across sexes and the skeleton; links between environment, husbandry, and transmission; the physical and ethological effects of ALV infection; the impact upon flock productivity; and the archaeological evidence for the temporal and spatial dimensions of the disease.

## Avian leucosis viruses

Avian leucosis viruses (ALVs), first established as a cause for tumour diseases in the early twentieth century (Ellermann & Bang, 1908; Rous, 1910), are members of the *Alpharetrovirus* genus of the family Retroviridae. Avian leucosis virus infections are still routinely observed in modern broiler and egg‐laying flocks of chickens (Gao *et al.,* 2010) and have a negative effect on productivity (Payne & Venugopal, 2000). Despite their pervasiveness and the economic importance of controlling avian leucoses, the resulting skeletal lesions have been discussed using variable terminology which has been debated since they were first medically described. ‘Sporadic diffuse osteoperiostitis’, a condition bearing a resemblance to avian osteopetrosis with regard to obliteration of the medullary (endosteal) cavity in avian long bones, was described by Pugh (1927), then referred to as ‘osteopetrosis gallinarum’ when identified by Jungherr and Landauer a decade later (Jungherr & Landauer, 1938). When it became evident that lesions affecting avians were aetiologically distinct from those affecting mammals and that characteristics such as the obliteration of the endosteal cavity developed secondarily, scholars began to suggest alternative terminologies. These were consistent with perceptions of viral infections at the time, with ‘transmissible multiple juvenile hyperostotic sclerosing osteopathia’ put forward by Thiersch (1948), ‘avian osteogenic osteoblastoma’ by Boyde *et al.* (1978), and ‘leukosarcomatous osteodysplasia’ most recently (Uzunova *et al.,* 2014). None has yet been fully embraced in non‐human palaeopathology, and ‘avian osteopetrosis’ continues to be used to describe bony lesions of the avian skeleton from ALV infection; it is favoured by most archaeological specialists, with some exceptions (e.g. Fabiš, 1997). The archaeological application of this term has resulted in a view of the disease as a simple viral infection, with little consideration for the relevance of environment, husbandry practices, or the impact of infection on productivity. In fact, such lesions reflect infection by a possible range of viruses in a series of subgroups (A‐J, of which some are endogenous and J is recently emergent) rather than a simple single agent (Payne, 1992; Vogt, 1977). Their prevalence in modern populations is firmly linked to the environment in which flocks are raised (Uzunova *et al.,* 2014), and husbandry methods are therefore a key component of pathogenesis.

Avian leucosis viruses vary in their ability to induce avian osteopetrosis and the rapidity of skeletal lesion development; persistent viraemia (the presence of the virus in the bloodstream) is essential for lesion formation but does not guarantee it (Robinson & Miles, 1985). In an experiment inducing avian osteopetrosis in chickens using leukaemic blood from mammalian sources, Thiersch noted that the time required for skeletal lesions to develop was reduced by repeated transmission of the virus in question (Thiersch, 1948: 101, 108). Male birds have been shown to be more susceptible to the development of avian osteopetrosis (Holmes, 1961; Robinson & Miles, 1985; Robinson *et al.,* 1992). Table 1 shows the range of domestic and commensal species known to be vulnerable to ALV infection, namely members of Galloanserae and Columbea (Vogt, 1977 373–376; Payne, 1992; Weiss, 2006). Passerines (parakeets) were experimentally investigated but found to be resistant (Vogt, 1977, 314).

**Table 1:** Species affected by avian leucosis viruses and the viral subgroups to which they are susceptible, adapted from Table 5 in Vogt, 1977:375

Species affected	Subgroups affected by	Reference
Chicken (Gallus gallus)	A‐E	Payne, 1992; many others
Guinea fowl (Numida meleagris)	A‐E	Payne, 1992; Uzunova *et al.,* 2014
Turkey (Meleagris gallopavo)	Not B	Holmes, 1963; Weiss, 2006
Common quail (Coturnix coturnix)	E	Weiss, 2006
Japanese quail (Coturnix japonica)	Not B	Moscovici and MacIntyre, 1966
King quail (*Excalfactoria chinensis*)	F, G	Vogt, 1977
Bobwhite quail (*Colinus sp.*)	D	Payne, 1992
Pigeon (Columba livia)	D	Sarma *et al.,* 1969; Payne, 1992
Chukar (Alectoris chukar)	A	Payne, 1992
Duck (*Anas platyrhnchos*)	C	Payne, 1992; Pruková *et al.,* 2007
Goose (Anser anser)	C	Payne, 1992
Pheasant (*Chrysolophus sp., Lophura sp., Phasianus sp., Syrmaticus sp.*)	F, G, otherwise by species; not B	Payne, 1992; Weiss, 2006

Aside from experimental techniques, ALVs are transmitted by three methods: horizontal (direct or indirect contact); vertical (congenital from female to offspring); and genetic (viral genome transmission from parent) (Pruková *et al.,* 2007). Quantities of ALVs are shed by both oral and cloacal routes, with the highest viral concentration in faeces; exposed skin is the portal of entry most conducive to infection (Weyl & Dougherty, 1977). Experimental transmission research demonstrated that ALVs can survive desiccation for 105 days (Simpson & Sanger, 1968: 272). A study of infected chickens found that whilst three in four viraemic hens transmitted ALV to their chicks, one in 12 non‐viraemic hens also did so (Rubin *et al.,* 1961). Because viraemia is necessary for skeletal pathology, this suggests that hens without easily detectable lesions could continue to perpetuate transmission. Cross‐species transmission of ALVs does occur and turkeys, quails, pheasants, and pigeons have been investigated in this regard (Holmes, 1963; Sarma *et al.,* 1969; Weiss, 2006).

The vulnerability of any given individual to infection is determined by a host of factors, including nutritional and immune state, climate, genetic predisposition, age and sex, environment, and sanitation (Inhorn & Brown, 1997; Roberts & Manchester, 2007: 167).

## Description and differential diagnosis

**Figure 2:**
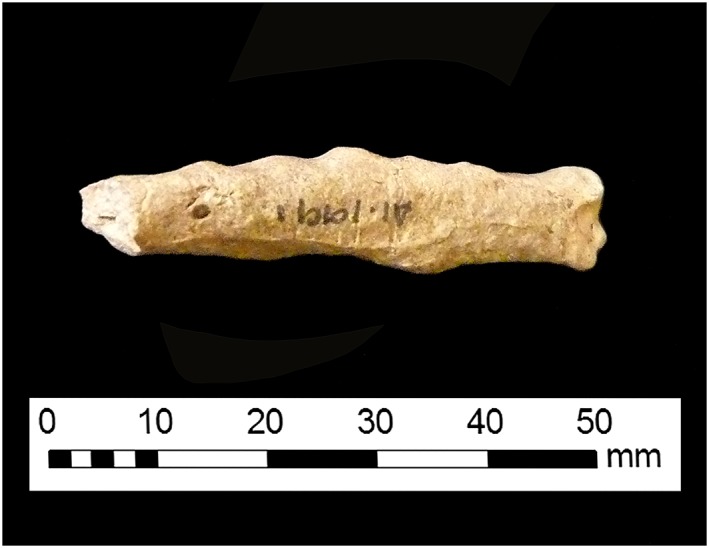
Chicken ulna from Causeway Lane in Leicester showing near‐complete occlusion of the endosteal cavity. [Colour figure can be viewed at wileyonlinelibrary.com]

Avian osteopetrosis lesions appear as hypermineralised osseous lumps projecting from the diaphyses of affected elements (Robinson *et al.,* 1992), which become thickened and expand periosteally and endosteally as total bone mass increases (Biltz *et al.,* 1965). Studies indicate that viral strains which cause a more rapid osteological lesion progression result in the formation of lesions with a rougher texture and appearance (Robinson & Miles, 1985; Simpson & Sanger, 1968). Complete obliteration of the endosteal cavity can occur (Figure 2), and increased radio‐opacity is universally evident (O'Connor & O'Connor, 2005: Figures 13 and 14). Lesions are consistently bilateral, and articular surfaces are unaffected (Simpson & Sanger, 1968; Thiersch, 1948).

Short‐term investigations (typically of less than a year in duration) of lesion development report that long bones are affected (Biltz *et al.,* 1965; Boyde *et al.,* 1978; Jungherr & Landauer, 1938; Simpson & Sanger, 1968; Thiersch, 1948), and that the tarsometatarsus (Simpson & Sanger, 1968), or the humerus, femur, or tibiotarsus are first to show lesions (Boyde *et al.,* 1978; Franklin & Martin, 1980). However, Holmes's longitudinal study demonstrated that all elements of the skeleton showed radiographically identifiable changes, but that they were affected at different rates (Holmes, 1961). Furthermore, the humerus, radius, ulna, femur, and tibiotarsus showed signs of skeletal pathology first and concurrently (Holmes, 1961: 370), which may partly explain why these elements are more frequently identified archaeologically (Figure 7). One possible instance of archaeological avian osteopetrosis has been recorded in a cranium from Mogador, Morocco (Becker *et al.,* 2013: 68). This element is one of the last to show skeletal lesions, and if this individual was suffering from ALV infection, they would have survived for approximately six months based on the rate of progression documented by Holmes (1961: 368). It may be that individuals were culled or disposed of soon after showing signs of illness. Although many elements affected later in the disease progression (e.g. the axial skeleton, metacarpals, pedal and wing phalanges) are difficult to identify to taxon and vulnerable to taphonomic and recovery biases, avian osteopetrosis would increase their potential survivability (Bartosiewicz, 2008).

Avian osteopetrosis lesions have a characteristic appearance which should not be confused with rickets or osteomalacia (as bowing of the elements does not occur), angular deformities (which can affect articulations and are not typically characterised by extensive new bone formation), or chondrodystrophies (which do not affect diaphyseal width). Trauma with extensive callus formation can be more difficult to eliminate by macroscopic inspection. Both avian osteopetrosis and callus formation involve endosteal and periosteal new bone proliferation and can be accompanied by element foreshortening. However, angular deviation from the natural axis of the element is not typical of osteopetrotic elements, and it does not affect articulations. Additionally, a fracture line is not present in avian osteopetrosis lesions but is diagnostic of some forms of trauma and can be detected using radiography (O'Connor & O'Connor, 2005: Figure 9). Despite these distinctions, some authors have excluded avian osteopetrosis from differential diagnosis based upon the assumption that ‘circumferential deposition of bone around the entire diaphysis’ is the defining characteristic of the condition (Cubo *et al.,* 2015: 9). Although this can occur, previous radiographic studies have demonstrated that avian osteopetrosis lesions are not consistently circumferential, especially in early stages (Holmes, 1961; Thiersch, 1948). Radiography is therefore key in attempting differential diagnosis of avian osteopetrosis.

Ethological and external physical changes have also been documented in birds infected with ALVs, as set out in Table 2.

**Table 2:** Ethological and externally observable physical changes in chickens which result from avian leucosis virus infection

Behaviour	External physical changes	Reference
Lethargy (30% of infected birds)		Uzunova *et al.,* 2014: 188
Anxiety/depression (20% of infected birds)		Uzunova *et al.,* 2014: 189
Cannibalism		Watts & Smith, 1980: 504
	Lameness or leg deformity sufficient to encumber movement	Uzunova *et al.,* 2014: 188–189; Robinson & Miles, 1985: 131
	Failure to develop sex characteristics, e.g. ‘infantile testicles’ in males	Holmes, 1961: 24
	Anaemia, ‘pallid yellow cast to legs and combs’	Holmes, 1961: 24; Paterson & Smith, 1978: 891
	Stunting	Holmes, 1961: 24–25; Hirota *et al.,* 1980: 929; Banes & Smith, 1977: 876–884
	Area of lesions hotter than surrounding tissue/body temperature	Simpson & Sanger, 1968: 274, 277
	Limb paralysis	Robinson *et al.,* 1992: 870
	Emaciation or low body weight	Uzunova *et al.,* 2014: 188–189; Hirota *et al.,* 1980: 932; Smith & Morgan, 1982: 492
	Reduced growth rate (26% of control rate)	Banes & Smith, 1977: 876–884
	Diarrhoea	Pruková *et al.,* 2007; 15
	Delayed sexual maturation in females	Payne, 1992: 338 (citing Gavora *et al.,* 1980)
	Reduction in laying	Payne, 1992: 338 (citing Gavora *et al.,* 1980)
	Increased eggshell fragility	Payne, 1992: 338 (citing Gavora *et al.,* 1980)
	Decreased fertility and hatchability of eggs	Payne, 1992: 338 (citing Gavora *et al.,* 1980)
	Decreased egg size	Gavora *et al.,* 1991

Many of these changes would have been apparent to observers of diseased flocks in the past, and people may have chosen to selectively dispose of infected individuals later recovered as associated bone groups (deposits of animal bone, sometimes articulated skeletons or portions thereof, hereafter referred to as ABGs following Morris, 2010) (Groot, 2008). These viruses would have had other meaningful impacts: productivity of affected flocks would have decreased, both with regard to egg‐laying and meat yield, making the disease a likely concern for those engaged in avian husbandry.

## Disease narrative

An extensive search of published and grey literature reports concerning archaeological chicken remains was conducted for this study: major journals, excavation volumes, non‐human palaeopathology literature, and other resources such as the Archaeological Data Service (ADS, hosted by the University of York). Additionally, further specimens were identified by the author and responses from analysts contacted in person or via the ZOOARCH e‐mail list over a period of 18 months (Table 3). Although there can be some confidence that the vast majority of known incidences have been collected in this search, there remains considerable potential for avian osteopetrosis lesions to remain unidentified in assemblages, particularly in regions where detailed recording of either pathologies or avian remains may not have been routine. Furthermore, chicken elements are small in size, and the identification of pathology in assemblages from unsieved sites could have been affected by this. Lesions identified by the author were recorded using a modified version of Vann & Thomas (2006), photographed, and subjected to digital radiography using a Xograph DRagon mobile x‐ray unit (52 KvP; 1.6 mAs; 0.025 s) for differential diagnosis. Metrical sex was not assessed due to the documented impact of avian leucosis infection on skeletal element length (Holmes, 1961: 24–25; Hirota *et al.,* 1980: 929; Banes & Smith, 1977: 876–884), and no spurs or medullary bone were observed by the author.

**Table 3:** Archaeological sites with reported finds of avian osteopetrosis, including suspected cases. The elements listed in bold font have been differentially diagnosed

Site name	Element(s)	Date (AD)	Reference
Sanctuary of Jupiter Heliopolitanus, Carnuntum‐Muhlacker, Austria	Not noted/accessible	100–410	Gál & Kunst, 2014
Tienen, Belgium	**Tarsometatarsus**	250–300	Lentacker *et al.,* 2004
Amiens (Rue Lavalard), France	Tibiotarsus	150–250	Meniel, pers. comm.,
Aunedonnacum, France	Femur	14–37	Lignereux *et al.*, 1997
Bondorf (Villa Rustica), Germany	Not noted/accessible	100–300	Kokabi *et al.,* 1994
Künzing, Bavaria	Coracoid	200–250	von den Driesch & Pöllath, 2000
Buda, Hungary	**ABG (sternum, humeri, ulnae, radius, femur and tibiotarsi)**	1247–1686	Gál, 2008
Intercisa, Hungary	**Tibiotarsus**	81–500	Gál, 2008
Apollonia‐Arsur, Israel	Femur	1265–1265	Pines, pers. comm.
Naples, Italy	Not noted/accessible	−400–1500	Albarella, pers. comm.
Mogador, Morocco	Cranium	1–400	Becker *et al.,* 2013
Dordrecht, Netherlands	Tibiotarsus	1400–1900	van Wijngaarden‐Bakker & Krauwer, 1979
Tiel‐Passewaaij, Netherlands	ABG (humerus, ulna, tibiotarsus, tarsometatarsus)	60–270	Groot, 2008
Velsen, Netherlands	Not noted/accessible	15–30	Prummel, 1987
Vicus Vitudurum (Oberwinterthur), Switzerland	Not noted/accessible	1–400	Morel, 1991
Troia, Turkey	Two tibiotarsi	1–500	Fabiš, 1997
1 Poultry, London, UK	**Tibiotarsus**	95–125	MOLA database; Morris, pers. comm.
2‐12 Gresham Street, London EC2, UK	Tibiotarsus	43–410	MOLA database; Pipe, pers. comm.
28 Park Street, London SE1, UK	Tibiotarsus	43–1750	MOLA database; Pipe, pers. comm.
35 Basinghall Street, London EC2, UK	Tibiotarsus	43–1750	MOLA database; Pipe, pers. comm.
Ashton Roman Town, UK	**Ulna**	43–410	Mahoney, pers. comm.
Blossoms Inn, 30 Gresham Street, London EC2, UK	Five elements, not further specified	200–400	MOLA database; Pipe, pers. comm.
Causeway Lane, Leicester, UK	**Ulna**	43–410	Connor & Buckley, 1999
Chester (Nicholas St. Mews), UK	**Femur**	1600–1800	Gordon, 2016
Cirencester, UK	**Tibiotarsus**	43–410	Strid, pers. comm.
Colchester, UK	**29 elements: 11 tibiotarsi, 10 humeri, four ulnae, two tarsometatarsi, one femur, one fibula**	40–400	Luff & Brothwell, 1993
Docklands Light Railway, UK	**Humerus**	43–1750	MOLA database; Morris, pers. comm.
Dudley Castle, UK	**Tibiotarsus**	1533–1647	Thomas, 2005
Fishbourne Roman Palace, UK	**Two tibiotarsi and a coracoid**	43–410	Fothergill and Best, In Prep.
Little Lane, Leicester, UK	Tibiotarsus	100–500	Gidney, 1989
London Bridge station, Jubilee Line extension, London SE1, UK	Tibiotarsus	140–250	MOLA database; Pipe, pers. comm.
Norwich (Barbican Well), UK	One element, not identified	1400–1600	Albarella *et al.,* 2009
Old Grapes Lane Site A, Carlisle, UK	Two tibiotarsi	43–100	Allison, 2010
Plantation House, 23 Fenchurch Street, London EC3, UK	Tibiotarsus	1220–1550	MOLA database; Pipe, pers. comm.
Princesshay, Exeter, UK	**Radius**	43–410	Fothergill and Best, In Prep.
Rochester Riverside, Kent, UK	Femur	150–400	Rielly, pers. comm.
Roman Southwark, Union Street, UK	Tibiotarsus	43–410	MOLA database; Pipe, pers. comm.
Scole‐Dickleburgh, UK	Two humeri	43–410	Baker, 1998
St Mary Axe, London EC3, UK	**Tarsometatarsus**	120–160	MOLA database; Morris, pers. comm.
Uley, UK	**Tibiotarsus**	43–410	Fothergill and Best, In Prep.

**Figure 3:**
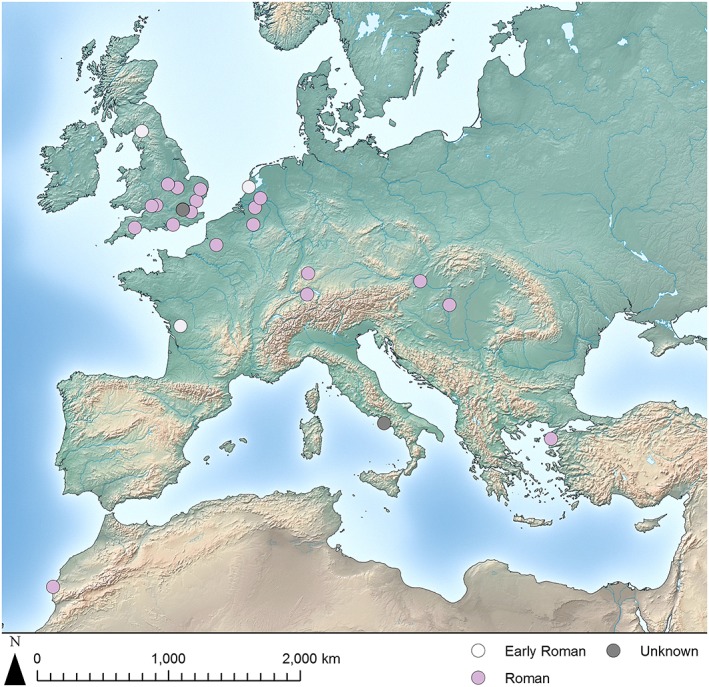
Map of Roman‐period archaeological sites with avian osteopetrosis lesions reported. [Colour figure can be viewed at wileyonlinelibrary.com]

**Figure 4:**
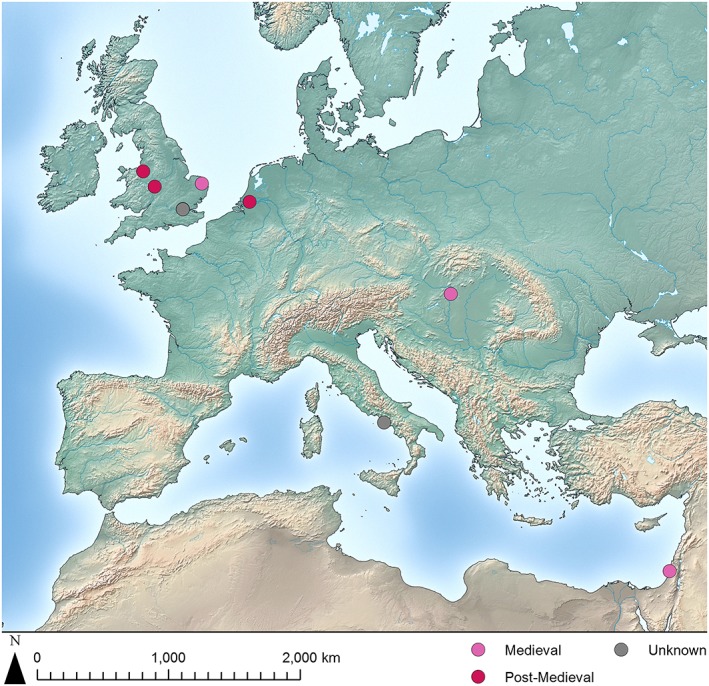
Map of Medieval and Post‐Medieval archaeological sites with avian osteopetrosis lesions reported. [Colour figure can be viewed at wileyonlinelibrary.com]

The geographic distribution of avian osteopetrosis lesions spans the Mediterranean and large swathes of Europe, with concentrations of sites near the Danube and the Rhine, in the Netherlands, and in the south of England (Figures 3 and 4). Excavation policies and faunal bone retention, analysis, and publication practices across this substantial area vary considerably and are likely to have affected this distribution. However, some spatial patterns are evident, particularly when temporal dimensions are also taken into account. The earliest instances of avian osteopetrosis (Figure 3, indicated in white) come from sites connected with the Roman military. Two have been identified in early first century AD (Tiberian) assemblages excavated from the fort at Aulnay in central France and the fort and naval base at Velsen in the Netherlands (Lignereux *et al.,* 1997; Prummel, 1987). Additionally, two possible instances of avian osteopetrosis from the first century AD were identified in the Old Grapes Lane assemblage from Roman Carlisle, UK (Allison, 2010).

The majority of sites with lesions described as avian osteopetrosis date to the first half of the first millennium AD. Most of these contexts have broad ranges, but none are definitively dated later than ad 250, and all are located within the boundaries of the Roman Empire at that time.

No lesions were identified in assemblages dating from the sixth to the twelfth centuries, but five sites have evidence of avian osteopetrosis from the Medieval and Post‐Medieval periods in areas near the Roman‐period distribution (Figure 4). Avian osteopetrosis lesions have been reported in modern Bulgarian flocks (Uzunova *et al.,* 2014), and the lack of sites from later Post‐Medieval contexts is probably related to the small, fragmentary nature of these assemblages and the lower perceived value of archaeological material from that time period (Thomas, 2009).

**Figure 5:**
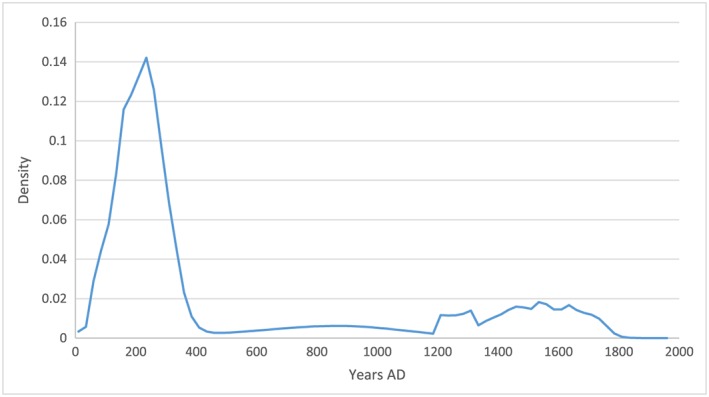
Linear distribution of the date midpoints of assemblages with avian osteopetrosis. [Colour figure can be viewed at wileyonlinelibrary.com]

Another visualisation of this data is provided in Figure 5, which shows the temporal spread of the date midpoints of assemblages with avian osteopetrosis using a linear distribution based on Willet's Gaussian method (Willet, 2014), smoothed using a 125‐year rolling average.

**Figure 6:**
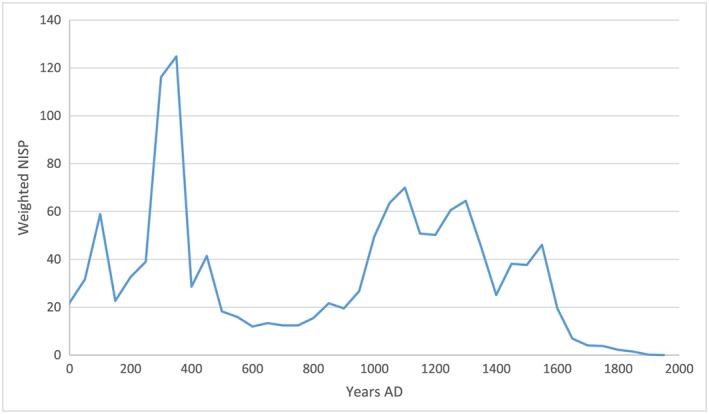
Linear distribution of chicken NISP weighted by date range of the assemblage. [Colour figure can be viewed at wileyonlinelibrary.com]

This distribution illustrates a trend for higher numbers of skeletal lesions in the first few centuries AD, which in turn suggests that flocks with viraemic individuals and higher rates of infection were more prevalent in the Roman period, coincident with the greatest extent of the Empire. A re‐emergence of the disease at densely‐populated urban sites is evident after the thirteenth century. Other factors are likely to have influenced this pattern, including fewer reported assemblages from the early medieval period and a drop in the presence and proportion of chickens in early medieval assemblages. For comparison, Figure 6 shows the distribution of identifiable chicken elements (NISP, weighted by the temporal range of the assemblage) over the same two millennia.

Based on data from Europe and North Africa collated from the Cultural and Scientific Perceptions of Human–Chicken Interactions project (2016) and other sources, the chicken NISP is highest at late Roman sites, which do not have correspondingly high lesion counts, and although the number of chicken elements increases just after ad 1000, avian osteopetrosis lesions are not identified with proportionate frequency.

Of the 74 archaeological chicken elements with avian osteopetrosis, none has been directly dated, and many have not been differentially diagnosed or described in detail (Table 3). Even so, the clustering of lesions in the first few centuries AD suggests that the disease spread rapidly across Europe, and that the movement of people and goods in relation to imperial Roman activity, especially given the presence of diagnosed lesions at sites along the *limes*, may have been a factor (Gál, 2008: 45 notes the spread of the disease in the Roman period). This has more recent historical parallels with the outbreaks of rinderpest in the eighteenth century, which took place in the context of increased movement of human and non‐human animals due to war, as well as reliance on cattle and cattle trading (Roeder *et al.,* 2013).

**Figure 7:**
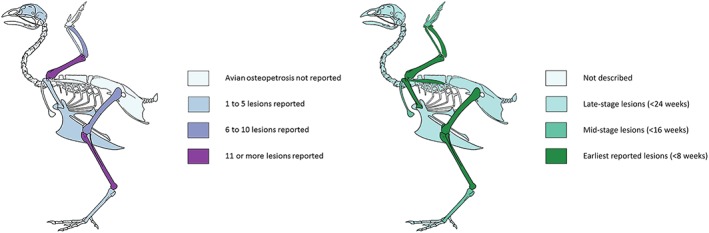
Skeletal diagram of archaeological elements with avian osteopetrosis lesions (left) and lesion progression according to Holmes (1961, right). [Colour figure can be viewed at wileyonlinelibrary.com]

Archaeological avian osteopetrosis lesions are not evenly distributed across the skeleton, and the most frequently affected element is the tibiotarsus, followed by the humerus. In addition to their high survivability, comparatively large size and the ease with which they can be identified, both of these elements are amongst the first to show skeletal lesions resulting from infection by ALVs (Holmes, 1961: 370). Figure 7 compares the archaeological elements affected by avian osteopetrosis lesions to the progression of lesions through the chicken skeleton (Holmes, 1961).

The elements initially affected during the progression of avian osteopetrosis are also those in which the condition is most frequently identified, which might suggest that the illness did not often progress beyond the initial skeletal stage and was terminated either by the effects of the virus or the actions of those responsible for the flocks. However, these elements are also large and survive both taphonomic and excavation processes reasonably well (Ericson, 1987); furthermore, they are often less complicated to identify to taxon than axial elements, phalanges, and ribs.

## Discussion

Without advanced genetic investigations and the application of direct dating methods to archaeological lesions, it is not yet possible to reconstruct the precise spatial and temporal origins of ALVs and therefore avian osteopetrosis, particularly as avian bone can be quite mobile with respect to archaeological stratigraphy (Lebrasseur *et al.,* In press). None of the earliest examples reported in this paper should be treated as a ‘patient zero’; indeed, the first cases of the disease may be much earlier. Instead, at some point in the early first century AD, ALVs took hold and spread widely within a few decades, undoubtedly the result of human action. Although archaeological perspectives on the movement of animal pathogens are rare, such activity has economic and social implications.

The appearance of avian osteopetrosis lesions in less suitable ecological contexts for keeping chickens may inform upon the spatial distribution. No lesions have been reported from Spain, Portugal, and most of North Africa (with the exception of Mogador on the west coast of Morocco), which are much warmer climes. Whilst the influence of under‐reporting cannot be ruled out for reasons discussed previously, the entirety of the Mediterranean offered a total of three assemblages with avian osteopetrosis present, and other factors may also be at work. Based on new ecological niche modelling using nine environmental variables (Pitt *et al.,* 2016), roughly half of the locations from which avian osteopetrosis lesions have been excavated are not suitable for chickens. The variables used included temperature seasonality, maximum and minimum temperatures, precipitation seasonality, maximum and minimum precipitation, maximum and minimum vegetation cover, and the availability of grit (Pitt *et al.,* 2016: 4). Chickens are descended from a jungle‐dwelling species, and most of Europe is not environmentally ideal, particularly with regard to low temperatures. It may be that the very measures which ensured flock survival and even marginal productivity (e.g. sheltering chickens or keeping them indoors) exacerbated the spread of ALVs by increasing chances of exposure. Conversely, the management of poultry species in areas which appear unaffected could have played a role in preventing transmission. Additionally, archaeological sampling, analysis, and publication policies with regard to faunal material vary widely and continue to evolve. It is likely that a combination of these factors has contributed to the geographic lacunae without archaeological evidence for avian osteopetrosis.

The frequency of sites related to the Roman military in northern and central Europe cannot solely be explained by ecological context and excavation strategies. The term ‘Roman military’ is used here in the broadest possible sense, to encompass the many distinct and diverse communities associated with it, rather than only soldiers (e.g. James, 2006). Movement of people and goods between Roman forts and their civilian settlements was routine and necessary. Although chickens are rarely mentioned in written sources, ALVs could have been transported in chickens or eggs as part of supply shipments (Erdkamp, 2002; Stallibrass & Thomas, 2008), or by soldiers and their families moving household or personal items (Allison, 2013: 20). Given that such viruses can withstand desiccation for more than three months (Simpson & Sanger, 1968: 272), and that the highest concentration of virus is shed in faecal material, even the surfaces of a cart, ship, or shoe could have played a role in spreading ALVs across the Roman Empire, especially considering the high value of chicken dung as fertiliser.

The osteopetrotic ABGs recovered from Tiel‐Passewaaij (Groot, 2008), Buda (Gál, 2008), and Colchester (Luff & Brothwell, 1993) may be the remains of individuals disposed of due to their diseased state. However, because non‐viraemic hens can pass ALVs to their offspring, even selective slaughter of birds with obvious symptoms would not have prevented perpetuation of the viruses responsible for avian osteopetrosis.

Chickens were introduced to Europe and North Africa in the first millennium BC, potentially via multiple east to west routes (Becker *et al.,* 2013; West & Zhou, 1988), and their remains are found archaeologically in contexts from across the study area by the second century BC. A programme of chicken element radiocarbon dating is currently being undertaken by Julia Best and colleagues, and a forthcoming publication will clarify the chronology of the introduction and early husbandry of the species. Although chickens were present in some Mediterranean regions for centuries before reaching northern Europe, the overall population appears to have boomed across the broad area considered here only in the first millennium AD.

Although poultry husbandry was practiced in diverse ways across the spatial and temporal extent considered here, Roman military sites warrant attention in this respect due to their strategic transport links and the presence of early avian osteopetrosis (although it is likely that the developed urban networks of the first centuries AD also played a role). The *pullari*, keepers of the chickens used in augury (often as part of military expeditions), are referred to by Cicero and Livy (*Cic. Fam.* 10.12.3, *Cic. Div.* 2.34; *Liv*. 8.30, *Liv.* 10.40, *Liv.* 9.14), and the Vindolanda tablets refer to chickens (and eggs) in the context of what appears to be a shopping list (*Tab. Vindol.* II 302), but their husbandry is not described. However, the management of other domestic animals is mentioned, and poultry keeping may have followed a similar practical arrangement. References are made to Alio, a veterinary doctor who would have been concerned primarily with equines (*Tab. Vindol.* II 181), those directly responsible for other domestic species such as cattle herders, and specific individuals named Lucco and Candidus, who were in charge of pigs (*Tab. Vindol.* II 180, 183). Prummel concluded that the chickens at Velsen were raised inside the camp, perhaps for cockfighting (Prummel, 1987: 186), and this may have been the case for other military sites. Also, given the importance of chickens at sites such as Uley and their dominance in assemblages from Mithraea (Lentacker *et al.,* 2004; Levitan, 1993; von den Driesch & Pöllath, 2000), it is also possible that the movement of birds destined for sacred roles increased the spread of ALVs. If the Roman military was a catalyst for technological change (as argued by Davies (2002) and others), and animal husbandry practices altered as a result, some changes may have fostered the emergence and transmission of viral disease. Given that the distribution of lesions is weighted towards the north‐west, we should keep in mind that military communities in the Roman world developed their own distinctive identities and practices. These trends may be the result of an intersection of different groups, environments, cultural practices, and diets that encouraged higher populations of chickens or the implementation of specific agricultural and husbandry practices that facilitated the spread of avian leucoses.

It is also probable that the drop in lesion frequency between the sixth and twelfth centuries was caused by multiple factors. A widespread decrease in the number and proportions of chickens in archaeological assemblages occurs across much of Europe, and this is likely to be linked to a lower number of reported assemblages from this period. The Medieval Warm Period also took place from *c.* ad 950 to 1250, temporarily altering the climate. Another possibility, especially as the later sites would have provided ideal conditions, is that this trend represents the cyclical re‐emergence of the viruses responsible, a process mired in complexity (Morens *et al.,* 2004). If this is the case, epidemiologic transition theory suggests that the sociocultural processes responsible for the pattern are diverse and challenging to frame (Barrett *et al.,* 1998). The subsequent rise in lesion frequency between the thirteenth and sixteenth centuries is of significant interest, but caution must be exercised as this trend is dependent upon a handful of specimens and is unlikely to have statistical validity. We can be confident, however, that husbandry practices had changed, although it is possible that the rediscovery of Roman agricultural writers and application of their methods during the Renaissance may have had an impact.

## Conclusions

This paper has considered the nature of ALVs, the bony lesions arising from infection, provisional differential diagnosis of lesions, and the role of human involvement, especially transportation of pathogens and application of husbandry methods, in the occurrence of avian osteopetrosis. Avian leucosis viruses were spread across Europe as a result of human action, likely connected in some way to the activities of the Roman military. The presence and progression of the infection are evident in affected individuals, some of whom may have been culled or otherwise disposed of due to their symptoms or behaviour. Apart from the usual biases affecting archaeological practice, ecological factors, cultural perceptions, and management techniques are likely to be partly responsible for the spatial and temporal distribution of avian osteopetrosis. The potential interpretive utility of diverse strands of evidence in non‐human palaeopathological investigation has also been demonstrated.

In addition to noting the impact of residuality, taphonomy, and archaeological practicalities (e.g. excavation, retention, analysis, and publication policies) on such findings, other factors should also be taken into account when constructing interpretations of past human‐animal relationships. Disease progression is often qualified (e.g. describing arthropathy as ‘advanced’), but other characteristics such as sex and individual or group ethology are intrinsically linked to processes of pathogenesis and transmission, and should be incorporated into analysis and interpretation of pathology whenever possible. The ways in which environmental influences relate to the presence, frequency, transmission, and movement of pathogens should also be considered. Finally, whilst pinpointing the first archaeological evidence for the emergence of ALVs is an endeavour of interest, future investigation of transmissible livestock diseases in the past should critically examine their impacts on past flocks, herds, and the lives of the peoples who kept them in the appropriate biological and cultural contexts.
